# SAFE TRANSITIONS: POST-ED DISCHARGE FROM A RURAL CRITICAL ACCESS HOSPITAL

**DOI:** 10.1093/geroni/igac059.1337

**Published:** 2022-12-20

**Authors:** Kristie Foster

**Affiliations:** Alice Peck Day Memorial Hospital, Lebanon, New Hampshire, United States

## Abstract

This talk will focus on leveraging the strengths of small rural communities to improve transitions of care post ED discharge. We moved beyond post discharge telephone calls, and partnered with already existing community based organizations. These collaborative partnerships enabled the use of standardized bundles of interventions targeting specific risks such as falls, depression, polypharmacy and the lack of documentation around goals of care.

